# Treatment of endometriosis in different ethnic populations: a meta-analysis of two clinical trials

**DOI:** 10.1186/1472-6874-12-9

**Published:** 2012-04-19

**Authors:** Christoph Gerlinger, Thomas Faustmann, Jeffrey J Hassall, Christian Seitz

**Affiliations:** 1 Global Biostatistics, Bayer HealthCare Pharmaceuticals, Müllerstraße 178, 13353, Berlin, Germany; 2 Global Medical Affairs, Women’s Healthcare, Bayer HealthCare Pharmaceuticals, Berlin, Germany; 3 Women’s Healthcare, Bayer (South East Asia) Pte, Singapore, Singapore; 4 Global Clinical Development, Bayer HealthCare Pharmaceuticals, Berlin, Germany

**Keywords:** Dienogest, Progestogen/progestin, Endometriosis, GnRH analogue, Ethnic differences, Europe/Japan

## Abstract

Approaches to the treatment of endometriosis vary worldwide, but studies comparing endometriosis medications in different ethnic groups are rare. A systematic literature search identified two studies directly comparing dienogest (DNG) versus gonadotropin-releasing hormone (GnRH) analogues in European and Japanese populations. Meta-analysis of visual analogue scale scores revealed no heterogeneity in response between the trials, indicating equivalent efficacy of DNG and GnRH analogues for endometriosis-related pain across populations. DNG was significantly superior to GnRH analogues for bone mineral density change in both trials, but significant heterogeneity between the studies may indicate ethnic differences in physiology.

## Background

Endometriosis is a chronic, painful disease caused by the growth of endometrial-like tissue outside the uterus, which induces a chronic inflammation resulting in fibrosis, adhesion formation, and scarring around the abdominal cavity and organs [1,2]. Endometriosis is common, occurring in approximately 10% of women of reproductive age [3], although the exact prevalence remains unknown because invasive procedures are required to confirm the diagnosis. Limited evidence suggests that the prevalence of endometriosis may vary with ethnicity. A slightly higher prevalence is reported in Japanese and other Asian women, and a lower incidence among African women, when compared to Caucasians [4-7]. Ethnic variations in prevalence could result from divergence in a range of genetic and environmental risk factors, which are thought to underlie the development of this complex condition [8-12]. Regardless of ethnicity, the symptoms of endometriosis are highly variable between individual women and often overlap those of other conditions. In many women, the symptoms are severe and quality of life can be greatly reduced [13-15].

Treatment approaches vary widely between regions worldwide, reflecting in part the availability of approved medications and divergences in surgical practice. In Japan, for example, gonadotropin-releasing hormone (GnRH) analogues appear to be widely used [16], while progestins account for a relatively small proportion of treatment regimens [17]. In other parts of the world, combined oral contraceptives and non-steroidal anti-inflammatory drugs are widely prescribed for relief of endometriosis-related pain, despite limitations in the supportive trial evidence, and the androgen, danazol, is still used in a number of countries despite its unpleasant side effects [18].

Progestins have been prescribed for the treatment of endometriosis for a number of decades, although the availability and actual use of approved progestins vary markedly between regions. The older progestins, in particular, may cause side effects (e.g. acne, hair growth and weight gain) that are associated with non-specific binding to androgenic and glucocorticoid receptors, and which may limit compliance with long-term treatment [19]. Newer generation progestins tend to have greater specificity for the progesterone receptor and offer favourable tolerability profiles [20,21].

Whether ethnic differences in response contribute to the variability in treatment approaches worldwide has been little studied. Research in this field is mainly restricted to small clinical pharmacology studies [22]. Large-scale clinical trials or meta-analyses comparing the efficacy and safety of medications in different ethnic groups are rare. Interestingly, two 24-week trials with similar designs were recently published comparing the efficacy and safety of the progestin dienogest (DNG) against GnRH analogues in European and Japanese women with endometriosis [23,24].

The aim of this paper is to provide a literature review of existing studies comparing DNG against GnRH analogues, and to explore, by meta-analysis, the results from these trials to investigate whether the treatment effects of DNG were similar or divergent between the European and Japanese populations.

### Treatments compared in this meta-analysis

The GnRH analogues are widely recognised as an effective treatment for endometriosis. Agents in this class act by modifying the release of follicle-stimulating hormone (FSH) and luteinising hormone (LH), thereby suppressing ovarian estradiol production. As endometriosis is an estrogen-dependent disease, this approach typically results in relief of symptoms; however, due to the potent suppression of endogenous estrogen production, GnRH analogues are associated with hypoestrogenic symptoms and loss of bone mass, limiting their use to short-term therapy or requiring add-back therapy [25].

DNG is a selective oral progestin that has recently received approval as monotherapy at a dose of 2 mg daily for the treatment of endometriosis in Europe, Japan, and other regions based on two independent, preclinical and clinical trial programmes conducted in Europe and Japan [26-33]. These clinical programmes included dose-ranging, placebo-controlled and active comparator-controlled studies of DNG, with study durations of up to 15 months.

### Literature search methodology

Based on an awareness of these studies, a systematic literature search was performed to identify potential additional relevant studies comparing DNG against GnRH analogues in endometriosis. A search of publications in PubMed using keywords including ‘endometriosis’, ‘dienogest’ and ‘gonadotropin-releasing hormone analogue or ‘GnRH analogue’ retrieved 11 publications [23,24,34-42], four of which were classified as ‘clinical trials’. Two of these trials were excluded from further analysis because they reported subpopulations of one of the remaining articles [41] or they included a different patient population by focusing on postsurgical consolidation treatment in women with endometriosis [39]. As a result, two trials qualified for inclusion in the meta-analysis.

A wider literature search for other approved medications in endometriosis identified no similarly designed, prospective trials that would permit a comparison of outcomes by meta-analysis in different ethnic groups.

The two DNG studies were both multicentre, randomised, 24-week trials to compare the efficacy and safety of DNG against a GnRH analogue in the treatment of endometriosis. Two hundred and fifty-two women from 17 centres in Europe were randomised to receive DNG (1 × 2 mg/day, orally) or leuprolide acetate (3.75 mg, 4-weekly intramuscular injection) [23], and 271 women from 24 centres in Japan received DNG (2 × 1 mg/day, orally) or buserelin acetate (3 × 300 μg/day, intranasally) [24]. Once-daily dosing of DNG was investigated in the European trial programme because of its potential for enhanced compliance. The European trial had an open-label design, while the Japanese trial was conducted double-blind.

Inclusion and exclusion criteria were broadly similar for both trials. Inclusion criteria included painful symptoms associated with endometriosis, confirmed by laparoscopy or laparotomy, in women aged 18 to 45 years (European trial) or aged 20 years or above (Japanese trial). Women who were pregnant or breast feeding, had used hormonal agents within specified times prior to the trial, had abnormal gynaecological examination findings (other than endometriosis) or who had risk factors for decreased bone mineral density (BMD) were excluded. Completion rates were greater than 85% in each treatment group in both trials [23,24].

Self-reported endometriosis-related pain (on a 0 to 100 mm visual analogue scale [VAS]) [43] was used as the primary assessment of efficacy in both trials, in line with current practice which recognises pain reduction as the most relevant treatment objective in women with endometriosis [44]. BMD (g/cm^2^) of the lumbar spine, measured by dual energy X-ray absorptiometry at selected study centres, was included as a safety outcome in both trials [23,24].

### Meta-analysis methodology

The current analyses focused on one quantitative measure of efficacy (i.e. VAS score) and one measure of safety (i.e. BMD), respectively. Efficacy analyses were performed on the primary per-protocol population in each trial (as appropriate for non-inferiority studies), and analyses of BMD included the eligible subset of women for whom data were available at both screening and final visit.

For each efficacy and safety outcome, the following methodology was employed. Changes in outcome from baseline to week 24 were combined for the two trials in fixed-effect meta-analyses, assuming an equal effect size [45]. The treatment difference between DNG and the GnRH analogue in each trial was used as the effect measure. The more powerful estimate of effect size of treatment gained from the combined trial results was used to extend and confirm the individual trial outcomes.

Heterogeneity across trials was described using the I² index [46] and tested using the Q-statistic [45]. The inverse variance method was used to weight the trials, based on the contribution of patient numbers and random variation (standard deviation [SD]). The two GnRH analogues studied were assumed to be equivalent to each other in efficacy and safety.

Statistical analyses were performed using version 2.10.1 of R software [47] and version 1.5.0 of the R software ‘meta’ package [48].

### Meta-analysis results

The women in the European and Japanese trials were of similar average age, while the European women had a slightly greater average body weight (Table 1).

**Table 1:** Baseline characteristics of the European and Japanese trial populations (full analysis sets)

	**European population **[23]		**Japanese population **[24]	
	**DNG (*n*** **= 120)**	**GnRH analogue (*n*** **= 128)**	**DNG (*n*** **= 128)**	**GnRH analogue (*n*** **= 125)**
Age (years, mean ± SD)	30.6 ± 6.2	31.0 ± 5.8	33.5 ± 6.9	33.8 ± 6.2
Weight (kg, mean ± SD)	62.5 ± 10.8	62.7 ± 9.6	52.1 ± 7.1	53.3 ± 8.2
Lumbar BMD (g/cm^2^, mean ± SD)	1.06 ± 0.1 (*n* = 26)	1.07 ± 0.1 (*n* = 31)	1.04 ± 0.1 (*n* = 41)	1.03 ± 0.1 (*n* = 46)

DNG, dienogest (2 mg/day); GnRH, gonadotropin-releasing hormone analogue; BMD, bone mineral density. GnRH analogues: European trial, leuprolide acetate 3.75 mg intramuscular injection, four-weekly; Japanese trial, buserelin acetate 3 × 300 μg/day, intranasally.

### Efficacy analysis (VAS change for endometriosis-related pain)

As previously reported, both trials concluded that DNG is non-inferior to the respective GnRH analogue for reduction of endometriosis-associated pain, based on VAS change.

**Figure 1:**
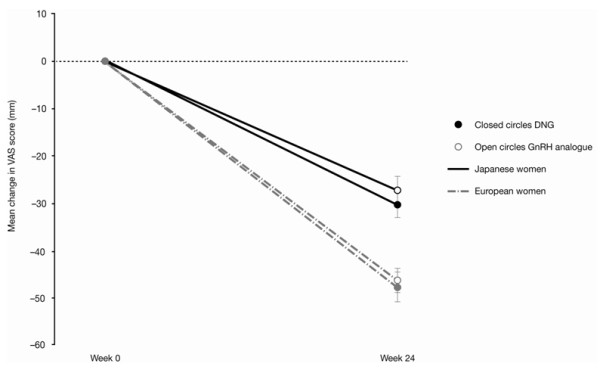
** Mean (± SEM) change in VAS (mm) between baseline and week 24 in the European **[23]**and Japanese **[24]**trials of DNG versus GnRH analogue.**

In the European population, VAS change for pelvic pain (mean ± SD) from baseline to week 24 was −47.5 ± 28.8 mm for DNG and −46.0 ± 24.8 mm for the GnRH analogue (Figure 1) [23]. In the Japanese trial, VAS change for lower abdominal pain from baseline to week 24 was −30.2 ± 31.8 mm for DNG and −27.3 ± 33.8 mm for the GnRH analogue (Figure 1) [24].

**Figure 2:**
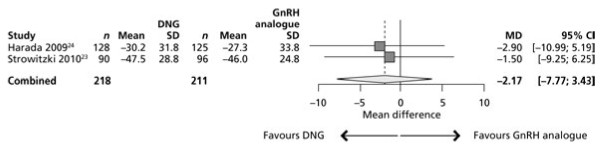
** Meta-analysis of change in pelvic pain measured on a VAS (mm) in the European **[23]**and Japanese **[24]**trials of DNG versus GnRH analogue.**

Mean treatment differences between DNG and the GnRH analogue were −1.50 mm (95% confidence interval [CI]: –9.25 to 6.25) in the European trial and −2.90 mm (95% CI: –10.99 to 5.19) in the Japanese population (both favouring DNG) (Figure 2). No heterogeneity between trial outcomes was apparent (Q-statistic = 0.06; *P* = 0.8065; 1 df; I² = 0%), suggesting that the effect of DNG for pain reduction, when compared with the respective GnRH analogue, was similar in the two populations.

Using the inverse variance method, the European trial had a weight of 52.17% and the Japanese trial had a weight of 47.83% in the meta-analysis. The lower weighting for the Japanese trial was due to a higher SD in VAS score change, despite providing 253 (57.63%) of the 439 women.

As the mean treatment difference in the Japanese trial was −2.90 mm compared to −1.50 mm for the European population, the inverse variance method for weighting provided a slightly smaller estimate of combined effect than weighting the trials equally. The combined estimate of the fixed effect model was −2.17 mm in favour of DNG (95% CI: –7.77 to 3.43) (Figure 2).

### Safety analysis (BMD change)

Both the European and Japanese trials reported that the study medications were generally well tolerated and that rates of premature withdrawals due to adverse events were low (≤ 5%). Hot flushes were more common in the GnRH analogue than the DNG group in both trials, while uterine bleeding was more common in the DNG group, as may be expected of a medication in the progestin class.

**Figure 3:**
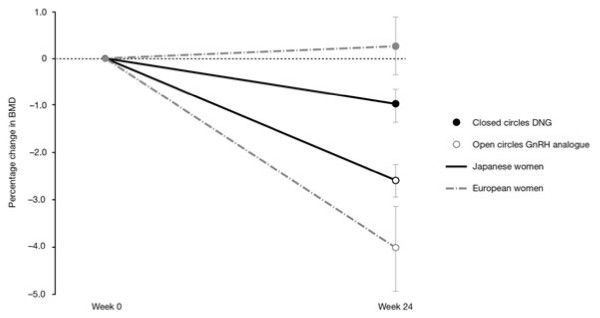
** Mean (± SEM) of percent change in BMD between baseline and week 24 in the European **[23]** and Japanese **[24]** trials of DNG versus GnRH analogue.**

DNG was significantly superior to the GnRH analogue with respect to change in lumbar BMD in both trials, i.e. causing no or less reduction in BMD [23,24]. In the European trial, in women whose measurements were available at both screening and final visit (*n* = 50), mean (± SD) BMD increased 0.25 ± 2.77% in the DNG group, versus a reduction of 4.04 ± 4.84% in the GnRH analogue group (*P* = 0.0003) (Figure 3) [23]. Respective changes in BMD in the Japanese trial were −1.00 ± 2.30% in the DNG group and −2.60 ± 2.30% in the GnRH analogue group (*P* = 0.003) (Figure 3) [24].

**Figure 4:**
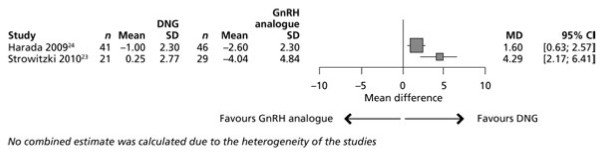
** Meta-analysis of per cent change in BMD in the European **[23]** and Japanese **[24]** trials of DNG versus GnRH analogue.**

The meta-analysis showed a treatment difference between DNG and the GnRH analogue of 4.29 (95% CI: 2.17 to 6.41) in European women and 1.60 (95% CI: 0.63 to 2.57) in Japanese women (both favouring DNG) (Figure 4). Significant heterogeneity between the trials was seen with respect to change in BMD (Q-statistic = 5.11; *P* = 0.0238; 1 df; I² = 80.4%).

The Japanese trial had a weight of 82.78% and the European trial had a lower weight of 17.22% due to the different sample sizes and variances (inverse variance method). No combined estimate was calculated due to the heterogeneity of the trials.

## Discussion

The global variability in standard approaches to the treatment of endometriosis could, in theory, reflect a number of influences, including divergent symptomatologies, variation in the range of approved medications available and variable treatment responses among different ethnic groups. The findings of our analysis indicate that responses to DNG, in terms of pain relief, are similar in the two populations investigated.

DNG had similar benefits for pain reduction after 24 weeks of treatment in each population, with mean VAS changes of −47.5 mm and −30.2 mm in European and Japanese women, respectively. The treatment group differences were −1.50 and

−2.90 mm in favour of DNG, respectively, in the individual trials, and were −2.17 mm in favour of DNG in the combined meta-analysis. When assuming a non-inferiority margin of 10 mm, as suggested by Gerlinger *et al*[43], these combined findings confirm the equivalent efficacy of DNG and GnRH analogues with regard to reduction of pain.

For the safety assessment, DNG showed significant benefits over the GnRH analogue in the change in lumbar BMD in each population [23,24]. These findings for DNG are in broad agreement with the results of Momoeda *et al*[29], who conducted a 1-year study of DNG treatment in a Japanese population. In this long-term study, a special focus was placed on BMD evaluation before, during and after treatment with DNG. BMD decreased by 1.6 ± 2.4% at 24 weeks and 1.7 ± 2.2% at 52 weeks, which the authors suggested reflects, in part, the normal decrease of BMD expected during ageing, with no relevant contribution from DNG [29].

Although DNG was significantly superior to the respective GnRH analogue for the change in BMD in both trials, there was heterogeneity between the trials in the size of this effect. This heterogeneity could be explained by a number of causes relating to ethnicity. The Japanese population had a slightly lower mean baseline BMD than their European counterparts (Table 1), in agreement with published data suggesting that Asian women have lower natural BMD [49]. Ethnic differences in physiological estrogen levels have also been reported, with young Asian women having higher concentrations compared with Caucasians [50]. Estrogen plays a role in the maintenance of bone health, and deficiency during either growth or ageing may lead to reduced BMD and increased bone fragility [51]. Compared to Caucasians, Asian women with estrogen deficiency are more likely to have BMD below the expected range for age [52]. Differing natural levels of estrogen throughout the lives of the European and Japanese populations studied may, therefore, have contributed to heterogeneity in the response to DNG and/or GnRH analogue treatment.

The two GnRH analogues investigated may also potentially be associated with differential effects on BMD. There appears to be no published literature that directly compares leuprolide acetate and buserelin acetate in endometriosis, but a review by the Cochrane Collaboration [25] suggests that, in similar trials, 24 weeks of treatment with buserelin acetate was associated with twice the BMD loss that was seen with leuprolide acetate [53,54]. However, these trials were conducted in different ethnic groups and the methods used for BMD analysis differed.

Limitations to this meta-analysis include the subtle differences in trial design (open-label versus blinded) and the different GnRH analogues (leuprolide acetate and buserelin acetate) used in the two trials. Additional information on demographics and lifestyle (dietary habits, level of exercise, etc.) of the participants may have provided further insights relevant to data interpretation.

## Conclusions

Treatment regimens for endometriosis vary widely between regions and it is unclear whether these different approaches reflect a difference in treatment response among ethnic groups. The availability of similar trials comparing DNG to GnRH analogues in European and Japanese women with endometriosis permits a comparison of the efficacy and safety outcomes in these ethnic groups. This meta-analysis suggests that women in Europe and Japan respond similarly in terms of pain relief to two of the main approved treatment options for endometriosis: DNG and GnRH analogues. Heterogeneity between the populations in BMD change requires exploration but may potentially be explained by differences in physiology or in response to therapy.

These results indicate that current differences in treatment patterns between regions may not be based on a medical rationale but on historical reasons or even chance. The findings support efforts to develop internationally agreed treatment guidelines in endometriosis that are applicable across populations.

## Competing interests

All authors are full-time employees of Bayer HealthCare Pharmaceuticals.

## Authors’ contribution

CG: development of study concept, statistical analyses, interpretation of study data, development of draft manuscript, review of advanced manuscript and approval of final version for submission. TF and CS: development of study concept, interpretation of study data, development of draft manuscript, review of advanced manuscript and approval of final version for submission. JH: development of draft manuscript, review of advanced manuscript and approval of final version for submission. All authors read and approved the final manuscript.

## Pre-publication history

The pre-publication history for this paper can be accessed here:

http://www.biomedcentral.com/1472-6874/12/9/prepub
